# Jewel in a Blouse

**Published:** 1861-10

**Authors:** 


### JEWEL IN A BLOUSE.

Passing along Sixteenth Street, some time ago, at the close of a Summer's day, we noticed a man walking before us in the commonest clothes of a laborer. He must have been over fifty years of age, and weary, too, for he walked with a weary gait, slow, and tottering. A few feet before him, near the centre of the smooth pavement, in front of Mr. Hoe's dwelling, there laid an ugly stone, not large, but just such an one as an unobservant, or old person, or little child, might stumble over. He took up the stone, carried it to the curb, laid it in the gutter, and passed along. "There's a grand heart in that poor little old man's body, in spite of its humble covering," said we to a lady with us. And it will be a lifelong regret to us that we did not overtake him and speak to him some word of cheer. That act was recognized in Heaven, for it was one of a pure benevolence, and it ought to have been recognized on earth; for, next to a good deed done, is its open, manly, and sympathizing approbation. This poor old man, in the lovingness of a kindly nature, without the stimulus of a present object of sympathy, which may move any one to help his brother, performed an act of only possible kindness, and that towards some unknown individual. It was a kindness to humanity in general.

Perhaps this incident may have made a deeper impression on us, from its having carried us back to our childhood; for one of its earliest memories is that of seeing our mother getting out of the carriage to remove some stones out of the road, which interfered with a clear way, the driver, meanwhile, looking complacently on from his seat. This act was one of spontaneous kindness, to save some after-comer, she knew not who, an uncomfortable jolt, or the annoyance of a break-down.

Within a few days, as if from inheritance, we found ourselves, before we were aware of it, removing a large stone from the centre of a narrow flag-way in the outskirts of the city. It must have been in that spot for months, for a smooth semicircular path had been made about it by the multitudes of feet which passed it daily; and yet, of all the crowds which thronged that way, for all that time, not a man, or woman, or child, had "moved away the stone."

Reader! what stones have you removed from the great pathway of life, with the wish, thereby, to remove hindrances to some after-coming brother? "What thou doest, do quickly," for "the time is short," and the day of life to many "is far spent."

